# Intra- and Interspecific Interactions as Proximate Determinants of Sexual Dimorphism and Allometric Trajectories in the Bottlenose Dolphin *Tursiops truncatus* (Cetacea, Odontoceti, Delphinidae)

**DOI:** 10.1371/journal.pone.0164287

**Published:** 2016-10-20

**Authors:** Maria Carla de Francesco, Anna Loy

**Affiliations:** Department of Biosciences and Territory, University of Molise, Fonte Lappone locality, Pesche, (IS) I-86090, Italy; Seconda Universita degli Studi di Napoli, ITALY

## Abstract

Feeding adaptation, social behaviour, and interspecific interactions related to sexual dimorphism and allometric growth are particularly challenging to be investigated in the high sexual monomorphic Delphinidae. We used geometric morphometrics to extensively explore sexual dimorphism and ontogenetic allometry of different projections of the skull and the mandible of the bottlenose dolphin *Tursiops truncatus*. Two-dimensional landmarks were recorded on the dorsal, ventral, lateral, and occipital views of the skull, and on the lateral view of the left and the right mandible of 104 specimens from the Mediterranean and the North Seas, differing environmental condition and degree of interspecific associations. Landmark configurations were transformed, standardized and superimposed through a Generalized Procrustes Analysis. Size and shape differences between adult males and females were respectively evaluated through ANOVA on centroid size, Procrustes ANOVA on Procrustes distances, and MANOVA on Procrustes coordinates. Ontogenetic allometry was investigated by multivariate regression of shape coordinates on centroid size in the largest homogenous sample from the North Sea. Results evidenced sexual dimorphic asymmetric traits only detected in the adults of the North Sea bottlenose dolphins living in monospecific associations, with females bearing a marked incision of the cavity hosting the left tympanic bulla. These differences were related to a more refined echolocalization system that likely enhances the exploitation of local resources by philopatric females. Distinct shape in immature versus mature stages and asymmetric changes in postnatal allometry of dorsal and occipital traits, suggest that differences between males and females are established early during growth. Allometric growth trajectories differed between males and females for the ventral view of the skull. Allometric trajectories differed among projections of skull and mandible, and were related to dietary shifts experienced by subadults and adults.

## Introduction

Since Darwin [1] sexual dimorphism and ontogenetic allometry have fascinated researches for their various evolutionary and ecological implications, ranging from utilization of different niches by sexes or age classes to avoid intraspecific competition, to the alteration of environmental and predation pressures, or to a combination of these factors [2, 3, 4, 5, 6, 7, 8, 9, 10]. Sexual dimorphism occurs when males and females of the same species differ in some morphological features, such as size (SSD—Sexual Size Dimorphism), shape, or secondary characters (i.e., colour, antlers, and mane). SSD is the most common phenomenon in mammals, being related to their polygynous reproductive strategy [11, 12, 13, 14, 15, 16]. In mammals, selection acts on resource optimization for pregnancy and lactation in females, while in males it favors the ability to monopolize access to females [17, 18, 19]. This latter phenomenon is expressed as either a resource defence polygyny or female (harem) defence polygyny, and it usually results in males being larger than females [18, 19]. Therefore, the vast majority of studies of sexual dimorphism in mammals have focused on SSD, while relatively few examined variation in shape [20, 21, 22, 23, 24, 25], especially in Cetaceans [26]. Odontocetes cetaceans (toothed whales) show different degrees of SSD, from the sperm whale having males 1.6 times larger than females, to the monomorphism of Delphinidae [11, 27]. A few taxa show secondary sexual traits, such as the beaked whales (*Ziphius cavirostris*), the killer whale (*Orcinus orca*), the narwhal (*Monodon monoceros*), or the fossil ziphiids [26, 28, 29, 30]. One of most challenging topic in evolutionary and conservation biology is to investigate if and how sexual dimorphism and ontogenetic allometry interact during growth in response to specific selective pressures [31, 32, 33, 34, 35]. This is critically important for understanding if and how resources exploitation varies according to sex or age, and how these intraspecific patterns are eventually affected by reproductive cycles or interspecific interactions [21, 26, 36]. This topic has not been extensively explored in Cetaceans, especially in Delphinidae [18, 19, 20, 21, 26, 37]. It is widely recognised that dolphins use echolocation to find their prey and for intraspecific communication, especially in socio-sexual relationships [38]. Specifically, information on sex and reproductive state of individuals interacting in a group is mediated by echolocation clicks and whistles produced and received through specific structures of the skull and the mandible [39]. As a consequence, the morphology of the mandible and the skull are not only related to prey catching and feeding adaptations [39] but also to echolocation performance, which in turn is strictly related to a strong directional asymmetry [40]. Thus investigating sexual dimorphism and ontogenetic allometry of dolphin skull and mandible may reveal specific adaptive traits and selective pressures related to reproductive strategies, intraspecific competition, or niche shifting during growth, and on how these factors interact during growth.

We specifically aimed to explore the evolutionary and ecomorphological significance of sexual dimorphism, ontogenetic allometry, and their interaction in the widespread bottlenose dolphin *T*. *truncatus* (Montagu 1821), using two-dimensional geometric morphometrics of the skull and the mandible. Although some authors reported differences in the number of teeth and height of the braincase of males and females of the bottlenose dolphin *T*. *truncatus* [41, 42, 43, 44], the role that sexual dimorphic traits and ontogenetic allometry play in the ecology of this species are currently unknown.

We particularly focused on the following questions: 1. Is sexual size monomorphism a constant trait of this species? 2. Is there any shape dimorphic trait of the skull or the mandible that could be related to any differential behaviour or adaptation of females and males? 3. Is there any geographic variation in sexual dimorphic traits that could be related to intra- or interspecific interactions? 4. Is there any allometric pattern that could be related to any adaptive shifting during growth?

## Materials and Methods

We investigated 104 sexed specimens of bottlenose dolphins (55 males and 49 females; 11 sub-adults and 93 adults) from ten European museum collections (Table 1).

**Table 1:** Sample information, listed for males (M) and females (F), sub-adults (S) and adults (A). l = left, r = right. Collections analysed: Institute Royal des Sciences Naturelles du Belgique (Belgium); Museo di Storia Naturale di Calci (Italy); Staten Naturhistorike Museum (Denmark); Fondazione Cetacea (Italy); Museo Civico di Storia Naturale Doria di Genova (Italy); Zoological Museum of Kiel (Germany); Museo di Storia Naturale di Milano (Italy); Naturalis Biodiversity Center Leiden (Nederland); Facoltà di Veterinaria dell’Università di Padova (Italy); Museo Civico di Zoologia di Roma (Italy); Museo di Storia Naturale La Specola (Italy) (S1 Appendix. Number and localities of specimens). Cranial projections as observed in Fig 1.

	Dorsal		Ventral		Occipital		Lateral l		Lateral r		Jaw l		Jaw r	
	M	F	M	F	M	F	M	F	M	F	M	F	M	F
**S**	5	5	4	3	5	5	5	4	5	5	6	5	6	5
**A**	47	44	42	35	45	44	48	44	47	44	44	38	43	39
**TOT**	**52**	**49**	**46**	**38**	**50**	**49**	**53**	**48**	**52**	**49**	**50**	**43**	**49**	**44**

**Fig 1:**
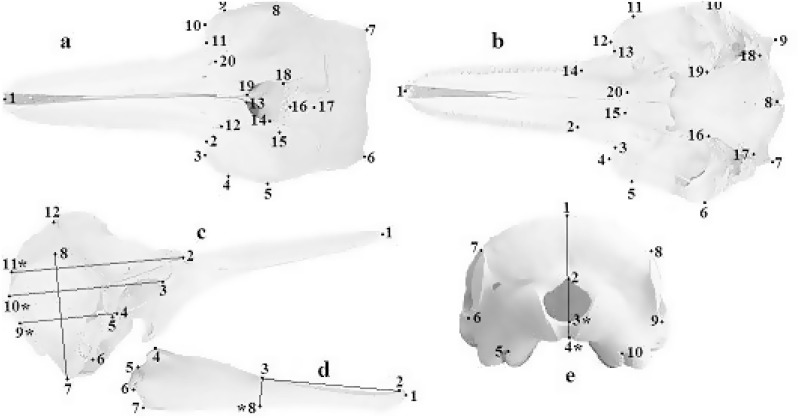
Dorsal (a), ventral (b), lateral (c), jaw (d), occipital (e) views showing two-dimensional landmark locations; (*) indicate derived landmarks. Landmarks 9, 10, 11 on the lateral view were identified as the points intersecting the perpendicular lines to the line connecting landmarks 7 and 8 from landmarks 2, 3, and 4, respectively. Landmarks 3 and 4 in the occipital view were identified as the points lying on the edge of the foramen magnum across the line joining landmarks 1 and 2. Landmark 8 on the mandible was derived by the line perpendicular to the line connecting landmarks 2 and 3.

Most specimens were strained along the coasts of the Mediterranean Sea (26 males, 16 females) and the North Sea (18 males, 17 females). These largest samples were respectively characterized by monospecific (North Sea) or interspecific (Mediterranean Sea) associations [45, 46]. The remaining samples were from the Atlantic Ocean (one female), Baltic Sea (one female), Pacific Ocean (four males, three females), aquaria (six males, 10 females), and two of unknown localities (one male and one female). Skulls were photographed in five standard projections: dorsal, ventral, occipital, left/right lateral; left and right mandibles were photographed in their medial projection. To minimize error due to image distortion, all skulls and mandibles were photographed at a fixed distance using a tripod and a Fujifilm Finepix S4000 camera set perpendicular to the specimen by means of a level. Specimens were considered osteologically matures (adults) when the maxilla and frontal bones were fused, and the maxilla and premaxilla were fused at the tip of the rostrum [31, 47, 48]. All other specimens were classified as immature (sub-adults). The software TPSUtil [49] was used to compile a database of images of the whole sample for each projection; 20, 20, 12, 10, and 8 two-dimensional landmarks were respectively placed on the dorsal, ventral, occipital, and left and right lateral view of the skull, and on the lateral view of the left and right mandible (Fig 1).

Landmark coordinates were digitized from images using TPSDig (version 2.17) [49] and then entered in MorphoJ (version 1.06b*) [50] for subsequent analyses (S1 Dataset). Original configurations were translated, rotated, and standardized to unit Centroid Size (CS), and optimally superimposed through a Generalized Procrustes Analysis (GPA) [51, 52, 53].

We first tested the effects of sex and association on skull shape variation of adult specimens. Sexual dimorphism was then further investigated through allometric growth trajectories of males and females subadults and adults. SSD was evaluated by univariate analysis of variance on log CS using the software PAST version 2.17c (Hammer and Harper 1999–2013). Shape variation was explored by Procrustes ANOVA and by multivariate analyses of the Procrustes coordinates with a Bonferroni correction [54], using MorphoJ.

Procrustes ANOVA was run considering effect of sex, association, and their interaction. The sample included 31 specimens (14 males and 17 females) from North Sea and Baltic Sea [21, 26, 45], known to live in monospecific associations (MA); 46 specimens (27 males and 19 females) from Mediterranean Sea, Tasmanian Sea, Japan, Perù, South Africa and Florida, establishing interspecific associations (IA) [46, 55, 56, 57, 58, 59, 60, 61, 62], and 14 specimens (six males and eight females) from aquaria (AQ). Specimens with undetermined location were excluded from the analysis.

Sexual dimorphism was then further investigated through allometric growth trajectories of males and females subadults and adults. Ontogenetic allometry was explored by multivariate regression of shape coordinates on log CS in all projections of the skull and the mandibles [63] in the largest homogenous dataset from one geographic area, i.e., the North Sea (18 males, 17 female; 6 sub-adults, 29 adults). Shape differences between age-classes (sub-adults versus adults) was explained though deformation grids of the skull and the mandible obtained by Relative Warps Analysis with TPSRelw software (version 1.11). Differences between males and females trajectories were analyzed by comparing the angles between regression vectors [64, 65] through the Angular Comparison function in MorphoJ [49, 66].

## Results

### Sexual dimorphism

ANOVA on log CS revealed no significant differences in the size of the skull between males and females of bottlenose dolphins (Table 2), confirming previous observations of a SSD monomorphism for this species [41, 44, 60, 67, 68].

**Table 2:** Procrustes ANOVA testing for shape and size differences between association, sex, and interactions between them (only adult specimens).

	Shape	Dorsal	Ventral	Occipital	Lateral l	Lateral r	Jaw l	Jaw r
**ASS**	**F**	3.34	1.93	2.08	3.35	2.48	3.78	9.08
	**df**	108	108	48	60	60	36	24
	***p*-value**	**<0.0001**	**0.0003**	**0.0062**	**<0.0001**	**0.0003**	**<0.0001**	**<0.0001**
**SEX**	**F**	1.55	1.40	0.94	1.34	1.24	0.31	1.74
	**df**	36	36	16	20	20	12	12
	***p*-value**	0.0537	0.0934	0.5302	0.1921	0.2554	0.9837	0.1199
**ASS xSEX**	**F**	1.36	1.28	1.39	0.96	1.59	1.17	0.83
	**df**	108	108	48	60	60	36	24
	***p*-value**	**0.0082**	**0.0307**	**0.0419**	0.5746	0.2900	0.2310	0.6975
	**Size**							
**ANOVA logCS**	**F**	0.00584	0.52	3,34	0.03	0.21	0.07	0.21
	**df**	91	76	88	91	92	83	82
	**p-value**	0.94	0.47	0.07	0.64	0.40	0.79	0.65

**Fig 2:**
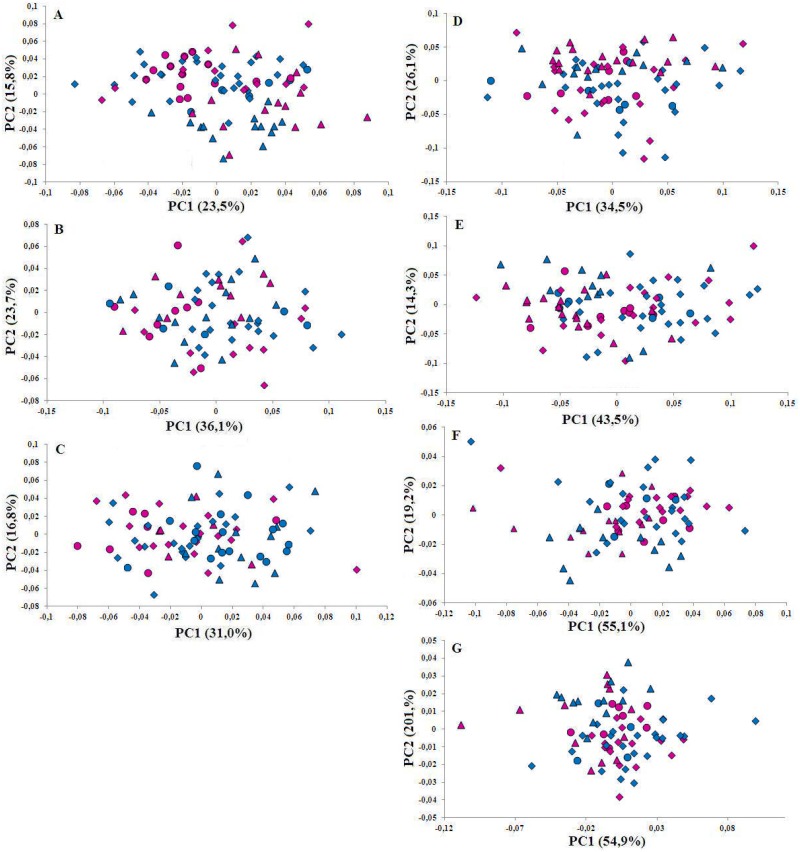
PC1 vs. PC2 scores of shape variables for dorsal (A), ventral (B), occipital (C), left (D) and right (E) lateral projections, and of left (F) and right (G) lateral mandible. Light blue symbols: males; pink symbols: females. Triangle: wild monospecific association; Rhombus: wild interspecific association; Circle: aquaria.

PCA and Procrustes ANOVA on shape variables of adult males and females revealed a significant distinction between populations of bottlenose dolphins living in mono- and multispecific associations (Table 2 and Fig 2A, 2B and 2C), and no differences between males and females in any projection of the skull and mandibles. However, significant differences were revealed in the dorsal, ventral and occipital projections of the skull when considering the interaction between association and sex (Table 2).

**Fig 3:**
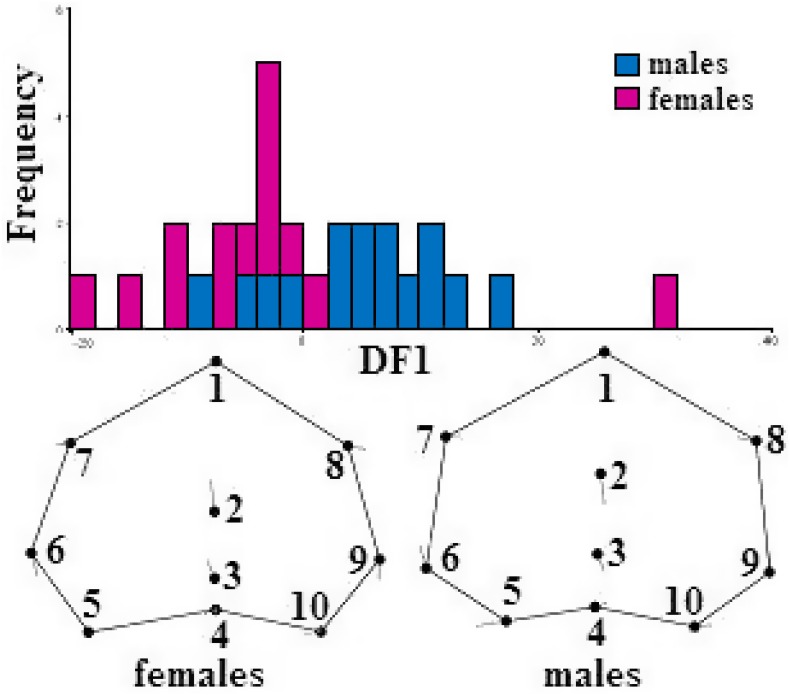
Top. Wireframes depicting shape changes at positive (left) and negative (right) extremes of the DF axis of males and females occipital shape coordinates. Vectors indicate landmark displacements (exaggerated by 5 points for better visualization).

A more detailed analysis of shape differences for the dorsal, ventral and occipital projections was then performed separately for the two largest populations of adult specimens living in monospecific and interspecific associations, i.e. respectively the North Sea (35 specimens; 17 females, 18 males) and the Mediterranean Sea (36 specimens; 16 females, 22 males). ANOVA, Procrustes ANOVA, and MANOVA of Procrustes coordinates confirmed a significant difference between sexes in the occipital projection of the North Sea population living in monospecific association (Table 3). Specifically, sexual shape dimorphism was evident in the degree of the asymmetry of specific occipital traits. That is, males and females differ in the magnitude and position of the incision of the basioccipital bone (landmarks 3, 5, 10 in Fig 3), corresponding to the cavity housing the left tympanic bulla. Differences are more marked on the left side (landmark 5 in Fig 3).

**Table 3:** Significance (probability values) of multiple regression of shape coordinates vs. log CS for the North Sea sample, and percentage of shape variance predicted by size for all projections.

	Dorsal	Ventral	Occipital	Lateral l	Lateral r	Jaw l	Jaw r
***p*-value**	**<0.0001**	**0.0165**	**<0.0001**	**<0.0001**	**<0.0001**	**0.0025**	**0.0002**
**% predicted**	8.3%	8.8%	7.5%	10.1%	15.5%	15.3%	16.9%

Fig 3 shows the cross-validation DFA scores of occipital shape variables for adult males and females from the North Sea, and the shape changes related to the extremes of variation along the axis (Mahalanobis distance = 3.3335, *p*-value = 0.0123).

### Allometry

Regression of shape coordinates of the North Sea sample onto log CS was significant for all of the projections, and shape variance predicted by size varied from 7.5% for the occipital view to 24.1% for the right lateral view of the skull (Table 3).

**Fig 4:**
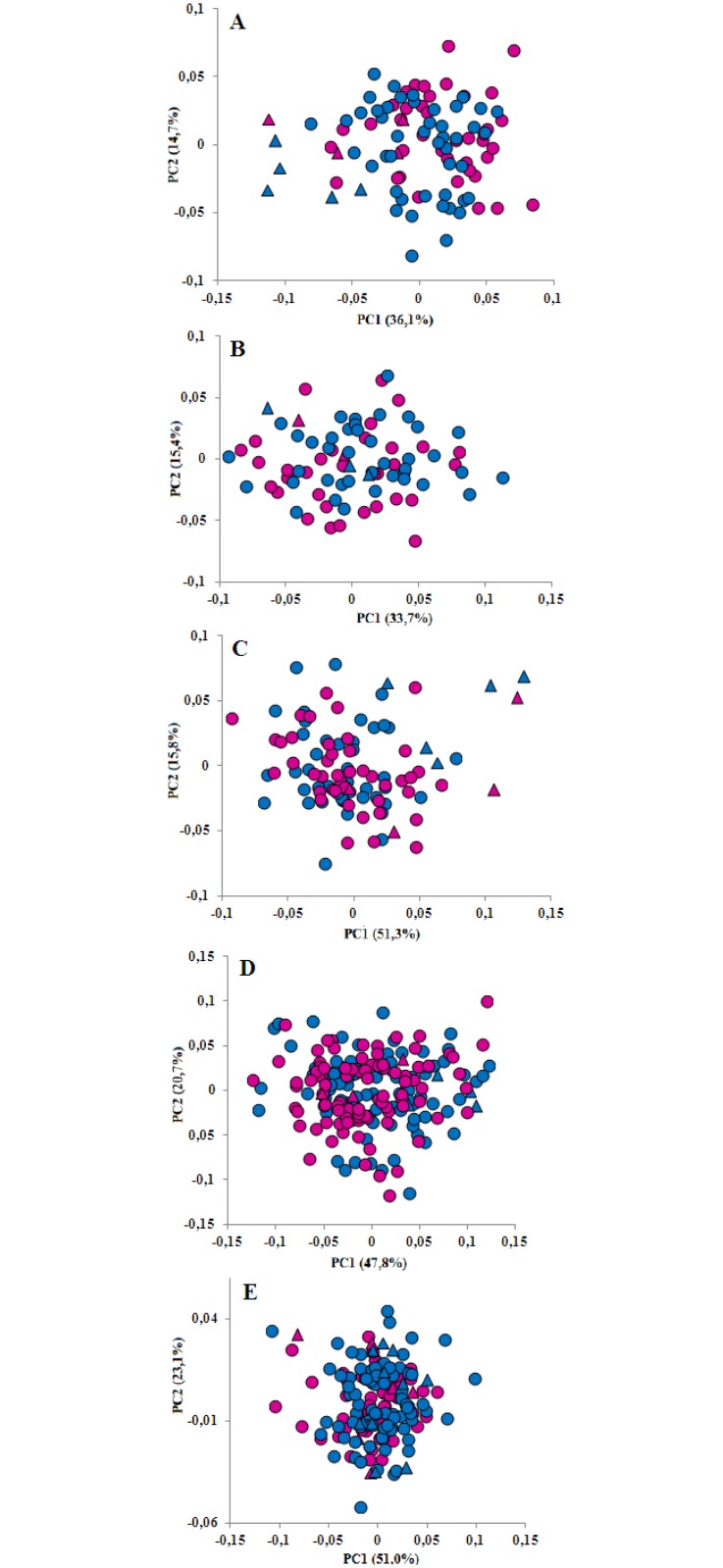
Scatterplot of PC1 vs. PC2 scores for the dorsal (A), ventral (B), occipital (C), left and right lateral (D) projections, and of left and right mandible (E) of sub-adult (triangle) and adult (circle) male (light blue) and female (pink) specimens from the North Sea.

The first two PC scores of shape coordinates showed a clear distinction of adults and sub-adults along PC1 for the occipital and the dorsal views (Fig 4A and 4C), while Procrustes ANOVA revealed that this distinction is significant for all projections but the ventral skull traits (Table 4)

**Table 4:** Procrustes ANOVA testing for shape differences between two age-classes (sub-adults vs. adults) in the North Sea sample.

	Shape	Dorsal	Ventral	Occipital	Lateral l	Lateral r	Jaw l	Jaw r
**AGE**	**F**	10.63	0.54	16.70	11.23	10.63	6.06	8.54
	**df**	36	36	16	20	20	12	12
	***p*-value**	**<0.0001**	0,9885	**<0.0001**	**<0.0001**	**<0.0001**	**<0.0001**	**<0.0001**

**Fig 5:**
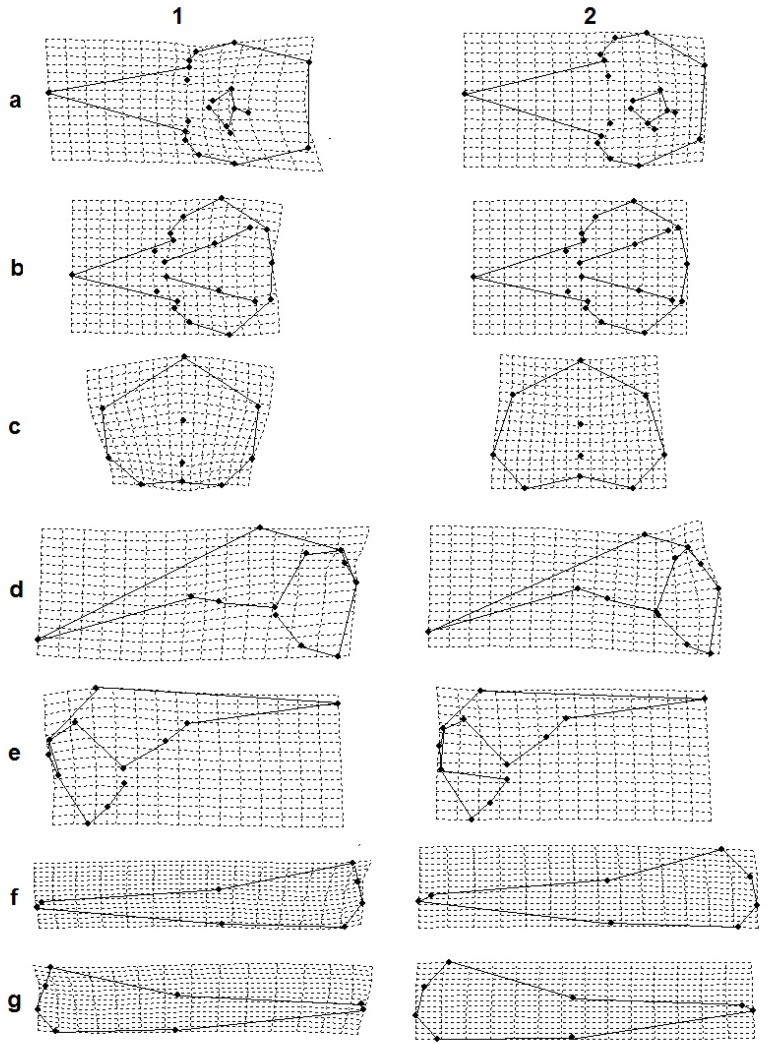
Deformation grids corresponding to the PC1-PC2 scores of the smallest sub-adult (CS = 34) (1) and the largest adult (CS = 76) (2), irrespective of sex. Skull: (a) dorsal, (b) ventral, (c) occipital, (d) left (e) right lateral skull; mandibles: (f) left and (g) right

From sub-adults to adults the skull undergoes an extension of the rostral and the nasal bones, a shortening of the braincase and an enlargement of the right frontal bones. Specifically, the braincase increases in height and becomes more curved in the posterior part. These changes are accompanied by a dorsal-ventral compression, a shrinking of the occipital area, and the development of the zygomatic processes (landmarks 4 and 5 in Fig 1C; Fig 5C). Moreover, an asymmetrical change occurs during growth, as the left nasal bones extends towards the anterior part of the skull while the shape of the right ones remain fairly unchanged (Fig 5A).

**Fig 6:**
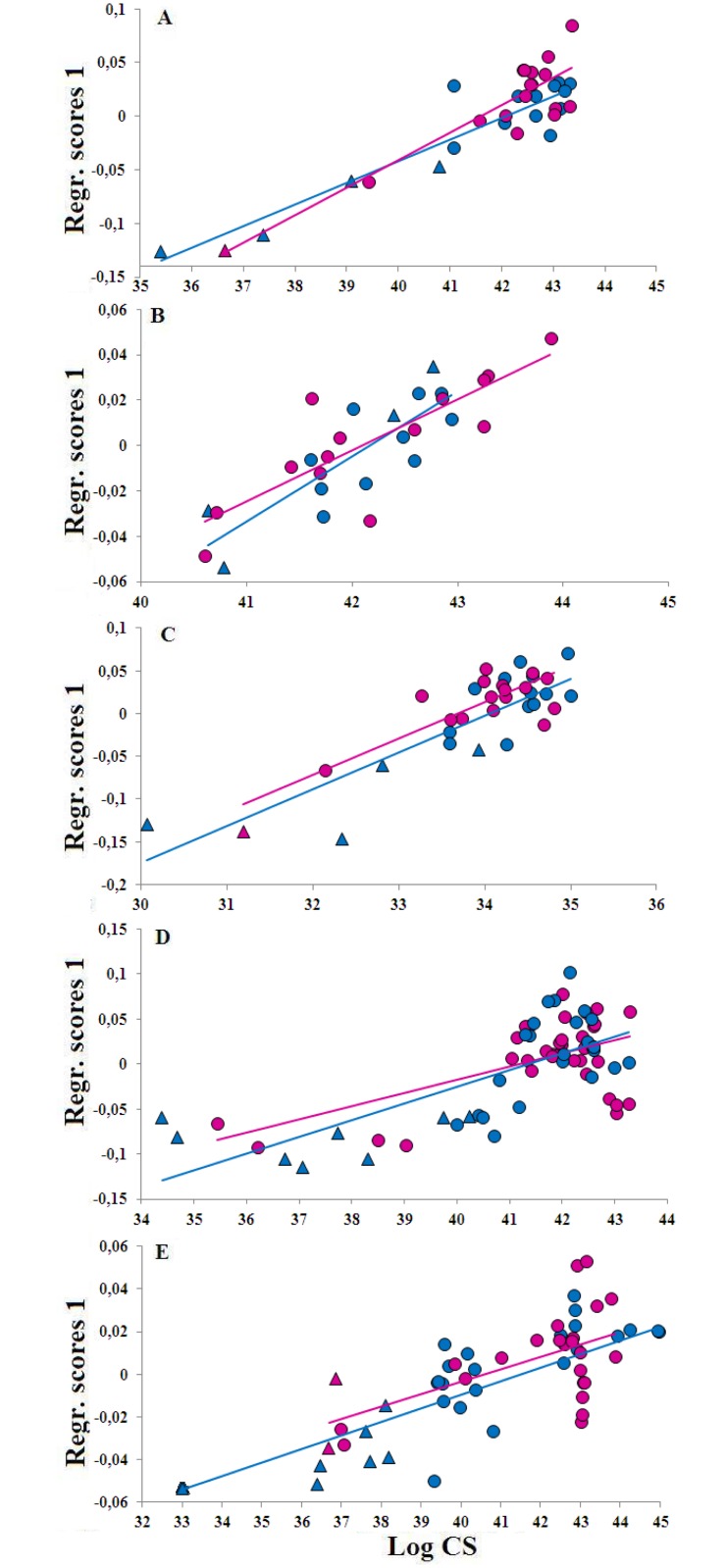
Log CS vs. the first multiple regression shape component of dorsal (A), ventral (B) occipital (C), and lateral projections (D) and of mandible (E) shape variables of the North Sea sample. Sub-adult: triangle; adult: circle; male: light blue; female: pink.

Allometric trajectories of males and females for all projections are shown in Fig 6. Both the dorsal and the ventral trajectories intersect (Fig 6A and 6B), suggesting that during growth the skull shape of males and females changes at different rates, with females showing the largest variability and the more extended growth. Interesting, in the ventral view the two age-classes are rather shuffled in size (Fig 6B). In the occipital view, allometric trajectories of males and females run in parallel (Fig 6C). The lateral skull showed a constant shape in sub-adults (from CS ca 30 to CS ca 55), followed by a sudden change in shape close to the adult phase (CS between 55 and 80), irrespective of sex (Fig 6D). The mandible trajectories showed a high degree of variability in both size and shape, and specimens appeared to be grouped into three distinct clusters, exhibiting a ‘step clined’ pattern (Fig 6E). This pattern suggests that the growth of the mandibles is characterized by an alternation of stasis and sudden shape changes, likely related to discrete age-classes adaptations. These shape changes mainly encompassed an increase in the relative length of the ramus and an enlargement of the corpus (Fig 6E).

To test the similarity between male and female trajectories we compared the angular vector directions in all projections of the skull and mandibles (Table 5) [64]

**Table 5:** Angle comparison between allometric vectors of males and females for all projections of the skull and mandibles. Significant values indicate similar trajectories [6,4].

Projections	Angle (in degree)	*p*-value
**Dorsal**	39.7°	**<0.0001**
**Ventral**	97.7°	0.7544
**Occipital**	19.7°	**<0.0001**
**Lateral**		
**Left** **right**	39.7° 29.8°	**<0.0001** **<0.0001**
**Mandible**		
**Left** **right**	16.9° 25.1°	**<0.0001** **<0.0001**

Results indicate that male and female trajectories are significantly similar for all the projections but the ventral skull. The trajectories of this latter view have an angle greater than 90°, thus excluding parallelism [64].

## Discussion

### Sexual dimorphism

Male and female bottlenose dolphins analysed in this work confirmed the sexual monomorphism in size that was observed in this species by many authors [41, 44, 60, 67, 68], and evidenced a sexual dimorphism in the shape of the skull only in the populations living in monospecific associations, especially in the North Sea sample.

Sexual size monomorphism is likely related to the peculiar reproductive strategy of the bottlenose dolphins. Female bottlenose dolphins are seasonally polyoestrous, producing 2–7 ovulations per year, and give birth to a single offspring after a 12 months gestation [58]. Cubs remain with their mother for several years, with an interbirth interval for females with surviving infants ranging from four to 15 years [69]. Females live in monosexual groups with their cubs [70, 71], but in contrast to many polygynous and sexual size dimorphic mammals, females groups are not monopolized by individual males [70]. Adult males typically associate in stable small groups that form consortships with single females with no signs of pregnancy, lasting from a few minutes to several weeks [69, 72]. This mating system has been associated to a mate coercion strategy [72, 73], even if it might not be the only mating strategy adopted by this species [72]. Multiple cycles confuse paternity and may allow females to retain choice of mating partners, rejecting at the next cycle an undesirable male for one considered better. This corresponds to a non-coercive breeding strategy [69, 74], and suggests that also mate choice with cryptic choice of sperm [75] might be an alternative mating strategy.

In this context selection on males should not act on adaptive traits that favour success in fights like the increase in size, but rather on traits that could either favour female compliance or choice. These traits could also be related to specific communication skills and therefore to specific traits of the brain, as males use vocal and physical threats to keep a female close during herding [69, 76, 77, 78, 79]. Further analyses are needed to eventually identify specific internal skull traits that could bear signature of such differences.

We did in fact found an evidence of sexual dimorphism in the shape of the skull, but only in the bottlenose dolphins that are known to live in monospecific associations, namely in the North Sea sample [21, 26], and not in the bottlenose dolphins living in interspecific association like the Mediterranean ones [46]. A geographic variation of sexual dimorphism in cranial shape was also found by [44] in bottlenose dolphins from the Gulf of Mexico and Florida, suggesting that differences in cranial shape between males and females might be not related to mating strategies, but rather to geographic variable factors, like interspecific associations, as suggested by some authors [47, 48, 80]. Dimorphic bottlenose dolphins from the North Sea likely experience a situation similar to that found by [81] in Virginia Beach and by [82] in the Moray Firth, Scotland. These authors observed bottlenose dolphins violently interacting with harbour porpoises, *Phocoena phocoena*, likely because of prey competition. In these areas, both species are sexually dimorphic in the skull [21, 26]. In such interspecific interactions, sexual dimorphism likely allows the separation of trophic niches between sexes, thus reducing the degree of intraspecific competition to compensate for the high interspecific one [4, 83]. Sexual dimorphism as response to a increased interspecific competition for feeding resources has been reported in many studies on birds and mammals [4, 8, 84, 85, 86, 87,88].

This hypothesis would also be supported by the low degree of sexual dimorphism or monomorphism shown by bottlenose dolphins associated with species that exploit different food resources, such as the association with *Globicephala* sp. in the Faroe Island and in the Strait of Gibraltar [89, 90], with *Stenella frontalis* in Florida [91, 92], and with *Delphinus delphis* and *S*. *coeruleoalba* in Santa Monica Bay, California [62, 93, 94]. Similarly, the absence of sexual dimorphism in the Mediterranean sample could be related to the association with *Delphinus delphis*, a species characterized by a different diet [46, 58, 95]. Specifically, females of the bottlenose dolphins from the North Sea showed a marked incision of the cavity hosting the left tympanic bulla. This asymmetric trait is likely related to differences in sound reception between females and males, rather than in the emission system. In fact, the melon, the structure responsible for sound emission, is located on the dorsal part of the cranium that did not exhibit any significant difference between sexes. A more marked incision of the cavity hosting the left tympanic bulla is probably related to a more refined echolocation system in female bottlenose dolphins. These differences might be related to the ‘bisexual philopatry’ [79] of the bottlenose dolphins, with females being more philopatric and males the more dispersive sex. Bisexual philopatry could result in sexual differentiation of the sensory structures where females become more specialized to exploit local resources. It is worth to note that tympanic bulla also differs in size in males and females of *Phocoena spinipennis* [96] and *Neophocaena phocaenoides* [97].

### Allometry

Significant allometric relationships involved all skull traits of the bottlenose dolphin, and the skull clearly showed a distinct shape in immature versus mature stages for all the projections of the skull but the ventral. In contrast, allometric growth was statistical different between males and females only for the ventral view.

Ontogenetic trajectories can differ in three ways: ontogenetic scaling indicative of change in the duration of growth, lateral shifts indicative of changes in prenatal development, and directional changes indicative of novel modes of postnatal growth [98, 99].

Postnatal allometry of dorsal and occipital traits of the bottlenose dolphins involved the relative extension of the rostral region and the braincase, and the shifting of nasal and right frontal bones. This latter asymmetric change might also be responsible for the higher directional asymmetry of the occipital bones in adult males and females from the North Sea. The strong directional asymmetry of the dolphin skull, always resulting in the enlargement of the right side of the skull, seems to be an adaptation to sound production and transmission [100]. Thus, the negative allometry displayed by the braincase could be explained by the asymmetrical growth of the melon in the left side of the skull that likely limits the expansion of the left frontal bones compared to the right ones [101].

Immature and mature stages did not show any distinct shape of the ventral traits. This constancy in the shape of the splancnocranium can be explained by the need to reach in a very early stage the final functional shape of the feeding apparatus, including the palate (palatine and pterygoid bones) and the base of the braincase (included the cavity housing of tympanic bulla), that is more stressed in males. Furthermore, the allometric trajectories of the ventral traits, i.e. the feeding apparatus, showed a more paedomorphic final shape in males [102]. Paedomorphic characters are rare in dolphins but common in the skull and the skeleton of porpoises [21, 103, 104, 105]. However, considering the small number of subadults that were available for this study, more evidences are needed to eventually confirm this hypothesis.

Peculiar allometric growth patterns were detected in the lateral view of the skull and in the mandible of the bottlenose dolphins, irrespective of sex. Sub-adults showed a ‘morphological stasis’ in the lateral skull shape until they reach a CS value of 60, followed by sudden morphological changes when approaching the adult stage (CS from 60 to 80). These sharp changes were marked by an enlargement of the braincase, evidenced by the expansion of the parietal and sub-occipital bones. In contrast, the growth of the mandibles was characterized by step clines and highly variable shape changes.

In general, the shape of the mandible showed an increase in its complexity, from an almost linear bar to a more complex shape involving the expansion and increase of the corpus, and a relative narrowing and curvature of the ramus (see Fig 5). A positive allometry of the rostrum was linked to the development of the zygomatic processes, where temporal muscles originate. The step clines could be related to a shift in diet and feeding behaviour from juveniles to the youngest and eldest adults. Specifically, the morphological step changes could be related to an increase in predation ability needed when shifting from small coastal prey caught by immature dolphins to large and pelagic preys caught by adults [27, 31, 70, 106].

As the shape of the dolphin mandible is also influenced by hearing functionality [68, 107, 108], its variation during growth could also be related to changes in sound reception and communication skills. This hypothesis could be supported by the different growth rates of the lateral projection of the skull and the mandible: the skull increased in size faster than the jaws during the first stage of growth, but the mandibles reached their largest dimension during the second stage, that is also accompanied by an increase in jaw complexity. Physiological researches revealed that the young bottlenose dolphins do not possess the same biochemical composition of acoustic window fat bodies (localized in distal part of the medial projection of the mandible) as adults, that during the growth change in weight, relative proportion and chemical composition [109]. The ontogeny of lipid accumulation could be related to the variation of mandible shape during the development of the individual and, consequently, could imply that young animals may not have the same ability to receive sound as adults.

We are aware that our results only represent a first insight into the sexual dimorphism and growth patterns of such complex and highly derived mammals as the Delphinidae, and that further data are needed to support any ecophyisiological hypothesis relating growth and sexual dimorphism with trophic resource exploitation and intra- and interspecific interactions.

## Supporting Information

S1 Appendix Number and localities of specimens. (DOC)

S1 Dataset 2D coordinates in TPS files. (ZIP)
