# Supplementary material for: Intra- and Interspecific Interactions as Proximate Determinants of Sexual Dimorphism and Allometric Trajectories in the Bottlenose Dolphin Tursiops truncatus (Cetacea, Odontoceti, Delphinidae)
Source: PLoS One. 2016 Oct 20;11(10):e0164287. doi: 10.1371/journal.pone.0164287 (PMC5072710; doi:10.1371/journal.pone.0164287)
Supplement: S1 Appendix — (DOC) [file pone.0164287.s001.doc]

**S1 Appendix**

Collections, codes, and geographic area of specimens analyzed

| **Collection** | **Total** | **Specimen codes** | **Macroarea** |
| --- | --- | --- | --- |
| **Institute Royal des Sciences Naturelles du Belgique (Belgium)** | 19 | 1517C, 1517D, 1517E, 16357, 17494, 17495, 17692, 18492, 18882, 18883, 20138, 20139, 20140, 20142, 20143, 39139, 40911, 32434, 33385. | North Sea  Monospecific Association |
| **Museo di Storia Naturale di Calci (Italy)** | 4 | 280, 281, 284, 285. | Mediterranean Sea  Interspecific Association |
| **Staten Naturhistorike Museum (Denmark)** | 22 | 2, 3, 4, 5, 6, 7, 8, 9, 10, 11, 12, 12bis, 13, 15, 22, 23, 24, 26, 27, 301, nocode, Anonimous. | North Sea  Monospecific Association |
| **Fondazione Cetacea (Italy)** | 14 | ER01, ER03, ER04, ER05, ER07, ER 09, ER10, LILLI, MA01, MA03, MA05, VE01, no code, Anonimous. | Mediterranean Sea  Interspecific Association |
| **Museo Civico di Storia Naturale Doria di Genova (Italy)** | 12 | 36405, 36410, 36412, 36413, 46867, 48564, 48569, 50246, 50249, 50250, 54707, 57160. | Mediterranean Sea  Interspecific Association |
| **Museo di Storia Naturale di Milano (Italy)** | 8 | 78, 470, 3968, 4900, 4902, 4919, 6694, 7279. | Mediterranean Sea  Interspecific Association |
| **Zoological Museum of Kiel (Germany)** | 8 | 1416, 1991, 1995, 2003, 2031, 2032, 2040, Anonimous. | Baltic Sea  Monospecific Association |
| **Facoltà di Veterinaria dell’Università di Padova (Italy)** | 16 | 71, 95, 96, 138, 139, 142, 146, 159, 162, 164, 165, 184, 185, 189, no code, Anonimous. | Mediterranean Sea  Interspecific Association |
| **Museo Civico di Zoologia di Roma (Italy)** | 9 | 30, 37, 39, 41, 42, 43, 44, 45, 46. | Mediterranean Sea  Interspecific Association |
| **Naturalis Biodiversity Center Leiden (Nederland)** | 83 | 933, 1466, 1558, 2113, 2247, 2330, 2450, 2484, 2721, 2725, 2943, 3155, 3313, 3351, 4515, 4900, 5072, 5873, 7316, 7651, 7893, 8157, 8158, 8159, 8160, 8161, 8164, 8617, 9128, 10504, 11764, 11807, 11952, 12593, 12597, 12896, 13190, 14595, 14596, 15383, 15384, 16162, 16455, 16853, 19146, 19785, 19799, 19827, 19837, 19872, 20295, 21173, 21227, 21331, 21349, 21420, 21452, 23027, 23868, 24128, 24677, 24678, 24679, 24680, 26121, 27044, 28061, 31185, 31186, 31187, 31188, 31189, 31190, 31191, 31192, 31193, 32350, 32351, 32352, 37957, 38137, 38328, Anonimous. | North Sea  Monospecific Association  Pacific Ocean  Atlantic Ocean  Interspecific Association |
| **Museo di Storia Naturale La Specola (Italy)** | 8 | 1597, 7176, 12604, 13348, 13350, 16573, 16606, 17833. | Mediterranean Sea  Interspecific Association |
